# Down-Regulation of Cannabinoid Type 1 (CB1) Receptor and its Downstream Signaling Pathways in Metastatic Colorectal Cancer

**DOI:** 10.3390/cancers11050708

**Published:** 2019-05-22

**Authors:** Valeria Tutino, Maria Gabriella Caruso, Valentina De Nunzio, Dionigi Lorusso, Nicola Veronese, Isabella Gigante, Maria Notarnicola, Gianluigi Giannelli

**Affiliations:** 1 Laboratory of Nutritional Biochemistry, National Institute of Gastroenterology “S. de Bellis”, Research Hospital, Castellana Grotte, 70013 Bari, Italy; valeria.tutino@irccsdebellis.it (V.T.); valentinadx@hotmail.it (V.D.N.); isabella.gigante87@gmail.com (I.G.); 2 Ambulatory of Clinical Nutrition, National Institute of Gastroenterology “S. de Bellis”, Research Hospital, Castellana Grotte, 70013 Bari, Italy; gabriella.caruso@irccsdebellis.it (M.G.C.); ilmannato@gmail.com (N.V.); 3 Surgery Unit, National Institute of Gastroenterology “S. de Bellis”, Research Hospital, Castellana Grotte, 70013 Bari, Italy; dionigi.lorusso@irccsdebellis.it; 4 Scientific Direction, National Institute of Gastroenterology “S. de Bellis”, Research Hospital, Castellana Grotte, 70013 Bari, Italy; gianluigi.giannelli@irccsdebellis.it

**Keywords:** endocannabinoid system, cannabinoid type 1 (CB1) receptor, colorectal cancer, metastasis

## Abstract

Changes in the regulation of endocannabinoid production, together with an altered expression of their receptors are hallmarks of cancer, including colorectal cancer (CRC). Although several studies have been conducted to understand the biological role of the CB1 receptor in cancer, little is known about its involvement in the metastatic process of CRC. The aim of this study was to investigate the possible link between CB1 receptor expression and the presence of metastasis in patients with CRC, investigating the main signaling pathways elicited downstream of CB1 receptor in colon cancer. Fifty-nine consecutive patients, with histologically proven colorectal cancer, were enrolled in the study, of which 30 patients with synchronous metastasis, at first diagnosis and 29 without metastasis. A low expression of CB1 receptor were detected in primary tumor tissue of CRC patients with metastasis and consequently, we observed an alteration of CB1 receptor downstream signaling. These signaling routes were also altered in intestinal normal mucosa, suggesting that, normal mucosa surrounding the tumor provides a realistic picture of the molecules involved in tissue malignant transformation. These observations contribute to the idea that drugs able to induce CB1 receptor expression can be helpful in order to set new anticancer therapeutic strategies.

## 1. Introduction

Cannabinoid receptors and their endogenous ligands, the endocannabinoids, constitute the endocannabinoids system (ECS), known to be important in regulating gastrointestinal motility, secretion, inflammation, and immunity [1]. ECS has been demonstrated to have a role in the regulation of signaling pathways involved in cancer pathogenesis [2,3]. Numerous studies have provided evidence that a deregulation of endocannabinoids production, together with an altered expression of their receptors are hallmarks of cancer [4,5,6]. The role of ECS in the onset of cancer is different and depends on the cancer type and tissue [6]. A high expression of Cannabinoid type 1 (CB1) receptor, but not cannabinoid type 2 (CB2), has been observed in pancreatic cancer [7], whereas an overexpression of both CB1 and CB2 receptors seems to be correlated with an improved prognosis in hepatocellular carcinoma [8]. In colon cancer, CB1 receptor has been detected at low levels and its inhibition has been demonstrated to accelerate intestinal adenoma growth [9]. In particular, CB1 deficient mice exhibited a more severe inflammatory status in the colon tissue, suggesting a protective role of this receptor against colonic tissue inflammation and malignant transformation [9,10]. Previously, we demonstrated a significant reduction in CB1 receptor gene expression levels in cancer tissue compared to normal surrounding mucosa of patients with colorectal cancer (CRC), confirming that the negative modulation of cell proliferation, mediated by CB1 receptor, is lost in cancer [11]. CB1 receptor induction seems to inhibit the proliferation of colon cancer cell lines, affecting cell cycle, in particular reducing the number of cells in the S phase and decreasing the cell polyamines content [12,13].

CB1 receptor gene up-regulation in Apc^Min/+^ mice fed a diet enriched with omega 3-PUFAs, was correlated with a significant inhibition of intestinal polyps growth mediated by a concurrent inactivation of the Wnt/β-catenin pathway [11].

The growth suppressing effect of CB1 receptor is due to a down-regulation of the epidermal growth factor receptor (EGFR) in prostate cancer cell lines [14] or via the activation of cAMP/protein kinase A pathway in breast cancer cells [15].

Experimental evidence suggests the use of cannabinoids as potential anticancer agents, given their ability to exert antitumoral effects in vitro and in animal models of cancer [11,12,13,14,15,16]. Cannabinoids via their receptors, especially CB1 receptor, can modulate signaling pathways that control cell survival and apoptosis [12,17]. Although several studies have been conducted to understand the biological role of the CB1 receptor in cancer, little is known about its involvement in metastatic process of CRC.

Metastatic CRC has limited treatment options and thus results in higher mortality rates compared to non-metastatic CRC [18]. The cancer cells that are able to survival in circulation, and to migrate and invade another organ, are characterized by the expression of cellular oncogenes or the loss of tumor suppressor gene function [19]. Identifying the cellular mechanisms that regulate metastasis onset may be useful to develop effective anticancer therapies.

It has demonstrated that endogenous ligands of CB1 receptor, as anandamide, are able to regulate several stages of the metastatic process, modulating both cell migration and invasion [20]. Cannabinoids seem also to have a regulatory role in angiogenesis, inhibiting the formation and tumor-induced angiogenesis in a model of endothelial tumor cells [21].

On the basis of these evidences and considering our previous data showing a significant reduction of CB1 receptor gene expression in cancer compared to normal surrounding mucosa from patients with CRC [11], here we hypothesize that CB1 receptor may be associated with disease severity of CRC. Therefore, the aim of the present study was to address a possible link between CB1 receptor expression and the presence of metastasis in patients with CRC. Moreover, we investigated the main signaling pathways at the basis of the action of CB1 receptor in colon cancer.

## 2. Results

Table 1 shows the clinical characteristics of 59 CRC patients enrolled in the study. The presence of synchronous metastasis was detected in 30 of 59 patients.

**Figure 1:**
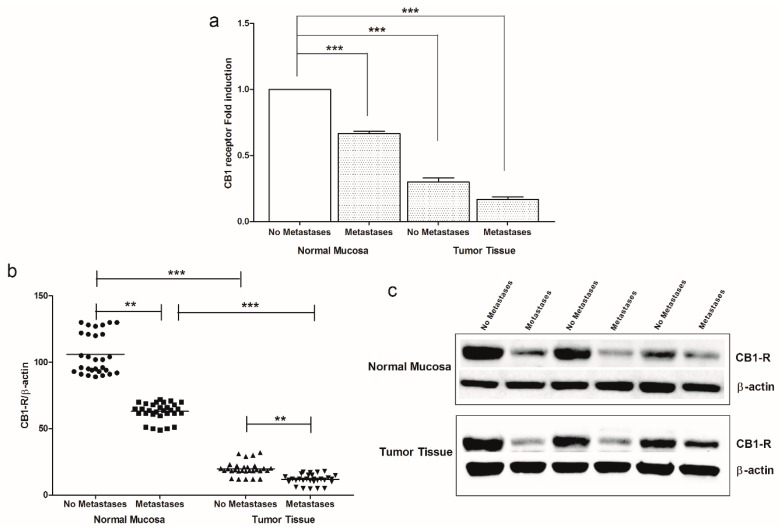
(**a**) CB1 receptor gene expression levels in intestinal tissue of no metastases (*n* = 29 patients) and with metastases patients (*n* = 30 patients) detected in both normal mucosa and tumor tissue. Data, expressed as mean value ± SD, are presented as fold induction compared to normal mucosa of patients without metastases. (**b**) Dot plots graph of CB1 receptor protein values detected in our patients groups. ** *p* < 0.02, *** *p* < 0.001 indicate statistically significant differences (one-way analysis of variance with Dunnett’s and Tukey’s multiple comparison test, where appropriate). (**c**) Representative Western blot bands of CB1-R and β-actin proteins. All Western blot figures include a dot plots graph showing the densitometry values of each sample (band) normalized to β-actin value. The whole blot has been provided as Supplemental Materials (Figure S1).

We detected a significant decrease in CB1 receptor gene and protein expression in colon tumor tissue compared to surrounding normal mucosa, confirming our previous data. Moreover, a significant down-regulation of CB1 receptor gene expression was detected in patients with synchronous metastasis, both in normal mucosa and in tumor tissue, as well as in tumor tissue of patients without metastases (Figure 1a). The CB1 receptor protein levels were measured using a specific CB1 receptor antibody [22,23], observing statistically significant differences of CB1 receptor protein expression between patients with and without metastases, both in intestinal normal mucosa and tumor tissue (Figure 1b,c). However, in normal mucosa, the levels of CB1 receptor protein were significantly higher than those detected in cancer tissue, suggesting that the protein expression was related to the ongoing neoplastic process.

**Figure 2:**
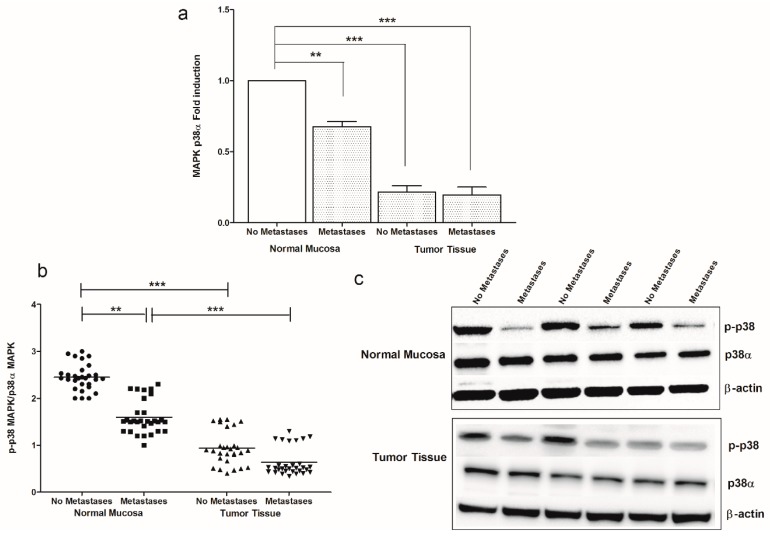
(**a**) MAPK p38α gene expression levels in intestinal tissue of no metastases (*n* = 29 patients) and with metastases patients (*n* = 30 patients) detected in both normal mucosa and tumor tissue. Data, expressed as mean value ± SD, are presented as fold induction, compared to normal mucosa of patients without metastases. (**b**) Dot plots graph of p-p38 MAPK/p38α MAPK ratio protein values detected in our patients groups. ** *p* < 0.02, *** *p* < 0.001 indicate statistically significant differences (one-way analysis of variance with Dunnett’s and Tukey’s multiple comparison test, where appropriate). (**c**) Representative Western blot bands of p-p38, p38α and β-actin proteins.

**Figure 3:**
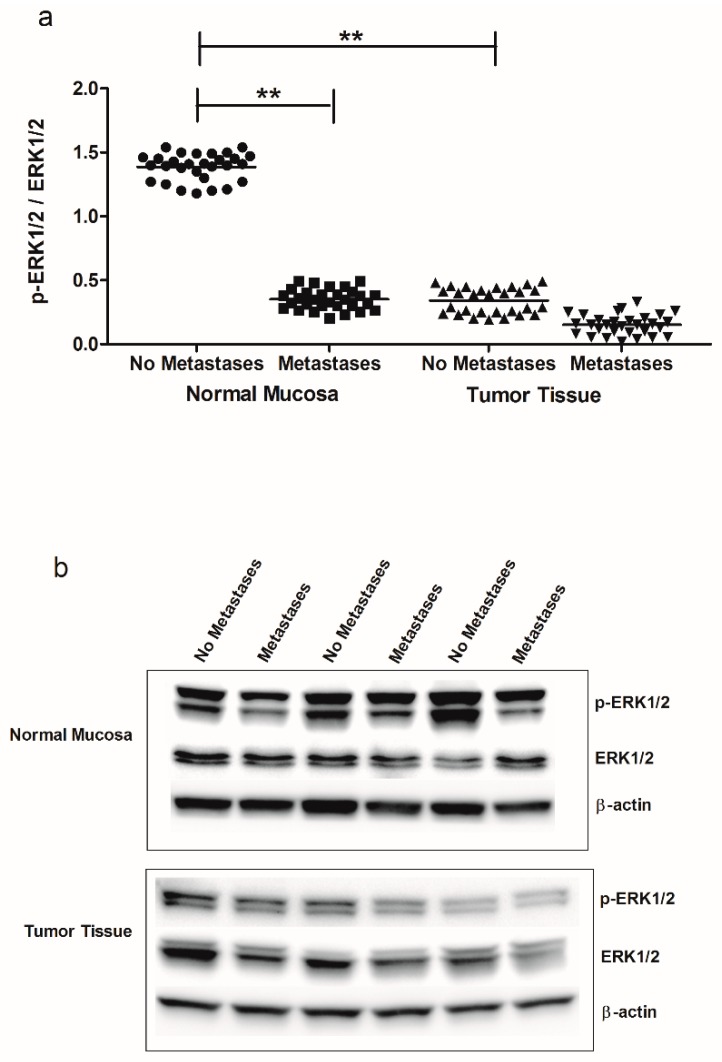
(**a**) p-ERK1/2/ERK1/2 ratio protein values detected in intestinal tissue of no metastases (*n* = 29 patients) and with metastases patients (*n* = 30 patients), in both normal mucosa and tumor tissue. ** *p* < 0.02 indicates statistically significant differences (one-way analysis of variance and Tukey’s multiple comparison test). (**b**) Representative Western blot bands of p-ERK1/2, ERK1/2 and β-actin proteins.

Down-regulation of CB1 receptor observed in metastatic CRC patients was linked to a decrease of downstream signaling such as the p38 mitogen activated protein kinase (MAPK) and extracellular signal-regulated kinase 1 and 2 (ERK1/2) pathway. Figure 2 shows the levels of mRNA (Figure 2a) and protein (Figure 2b,c) of p38 MAPK detected in normal mucosa and tumor tissue from CRC patients without and with metastasis. A statistically significant decrease was observed in patients with synchronous metastasis, both in normal mucosa and in tumor tissue, as well as in tumor tissue of patients without metastases, compared to normal mucosa from no metastases patients (Figure 2a). Statistically significant differences were also observed for p38 MAPK protein expression between normal mucosa and cancer obtained from patients with and without metastasis (Figure 2b,c). Figure 3 shows the western blotting analysis of ERK1/2 and p-ERK1/2 protein expression, demonstrating a significant decrease of p-ERK1/2/ERK1/2 ratio, overall in normal mucosa. Moreover, the patients without metastases showed low levels of p-ERK1/2/ERK1/2 ratio in tumor tissue compared to their corresponding normal mucosa (Figure 3a,b).

**Figure 4:**
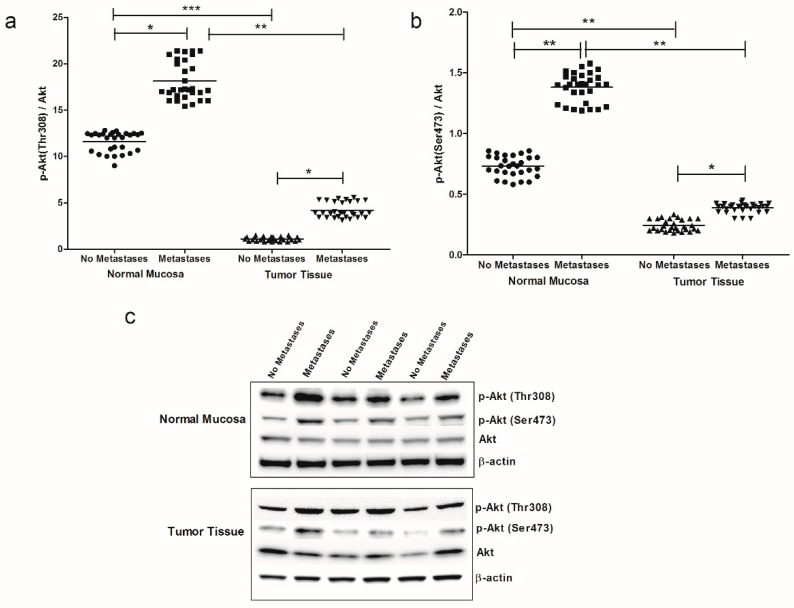
(**a**) p-Akt (Thr308)/Akt ratio protein values, detected in intestinal tissue of no metastases (*n* = 29 patients) and with metastases patients (*n* = 30 patients), in both normal mucosa and tumor tissue. (**b**) p-Akt (Ser473)/Akt ratio protein values detected in intestinal tissue of no metastases (*n* = 29 patients) and with metastases patients (*n* = 30 patients), in both normal mucosa and tumor tissue. * *p* < 0.05, ** *p* < 0.02, *** *p* < 0.001 indicate significant differences (one-way analysis of variance and Tukey’s multiple comparison test). (**c**) Representative Western blot bands of p-Akt (Thr308), p-Akt (Ser473), Akt and β-actin proteins.

Among the main signaling cascades elicited downstream of CB1 receptor action, we evaluated the protein levels of Akt, p-Akt (Thr308) and p-Akt (Ser473), observing higher expression of both p-Akt (Thr308)/Akt ratio and p-Akt (Ser473)/Akt ratio in tissue samples of CRC patients with metastasis compared to those from patients without metastasis (Figure 4a–c). This difference in p-Akt protein expression was evident both in intestinal normal mucosa and tumor tissue. Also for these proteins, the levels of expression were significantly higher in normal mucosa, compared to tumor tissue, demonstrating a greater impairment of cancer tissue.

**Figure 5:**
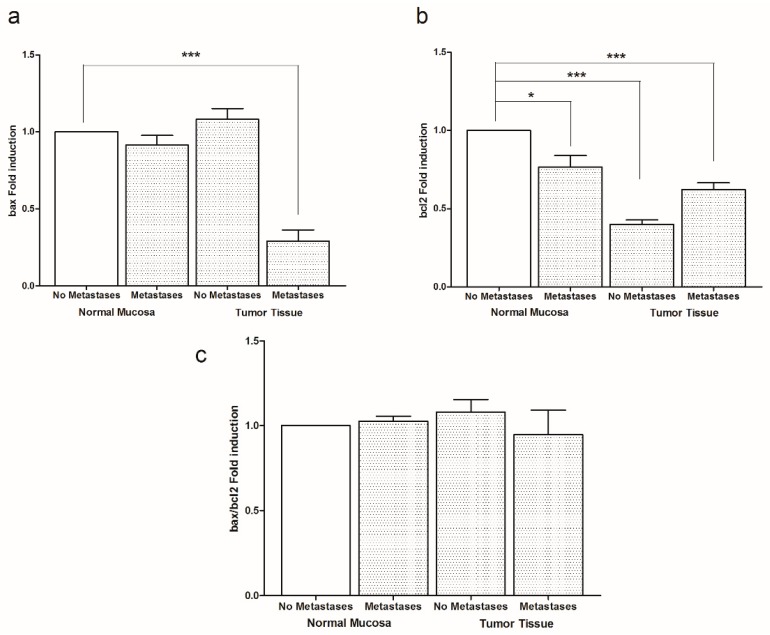
Transcriptional analysis of bax (**a**) and bcl2 (**b**) genes in intestinal tissue of no metastasis (*n* = 29 patients) and with metastasis patients (*n* = 30 patients) detected in both normal mucosa and tumor tissue. (**c**) shows the levels of bax/bcl2 ratio detected in the same intestinal samples. Data, expressed as mean value ± SD, are presented as fold induction compared to normal mucosa of patients without metastases * *p* < 0.05, *** *p* < 0.001 indicate significant differences (one-way analysis of variance and Dunnett’s multiple comparison test).

The presence of metastasis in our CRC samples was associated with an inactivation of apototic proteins. Figure 5 represents the mRNA levels of bax, bcl2 and bax/bcl2 ratio. A statistically significant reduction of bax gene expression was observed in tumor tissue from patients with metastasis (Figure 5a). For bcl2 gene, the levels of mRNA were significantly lower in all groups of patients (Figure 5b). Figure 5 panel c demonstrates that the presence of metastases exerted a reduction of bax/bcl2 ratio in tumor tissue, even if not statistically significant.

**Figure 6:**
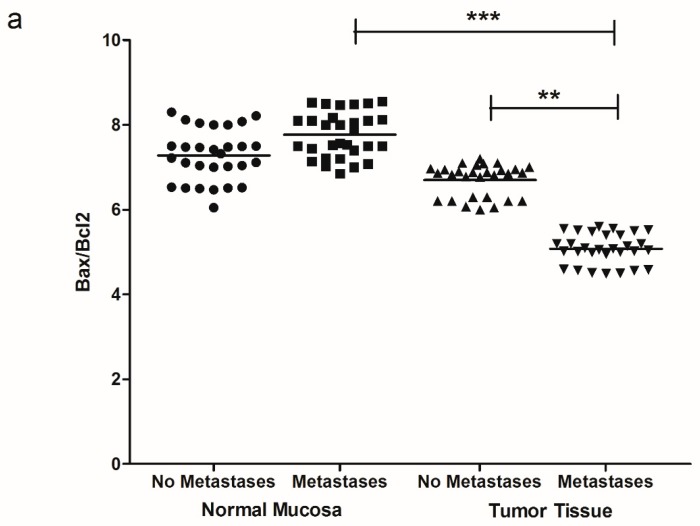

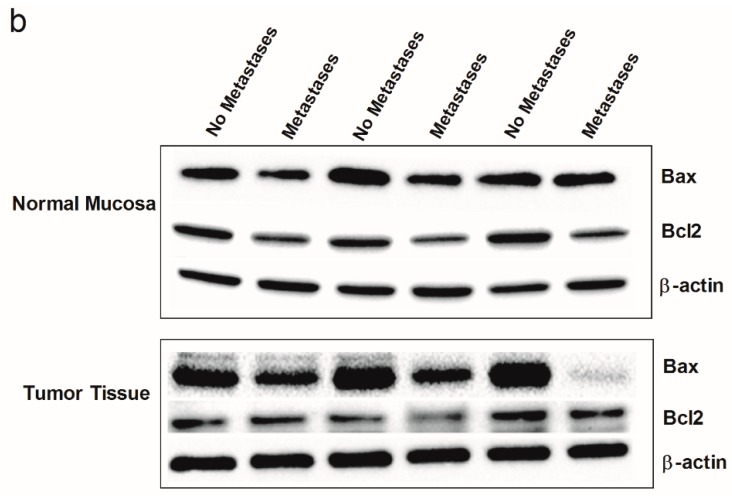
(**a**) Bax/Bcl2 ratio protein values detected in intestinal tissue of no metastases (*n* = 29 patients) and with metastases patients (*n* = 30 patients), in both normal mucosa and tumor tissue. ** *p* < 0.02, *** *p* < 0.001 indicate significant differences (one-way analysis of variance and Tukey’s multiple comparison test). (**b**) Representative Western blot bands of Bax, Bcl2 and β-actin proteins.

Figure 6 shows the protein expression of Bax/Bcl2 ratio, demonstrating a significant reduction of Bax/Bcl2 ratio in tumor tissue of the patients with metastases (Figure 6a,b).

**Figure 7:**
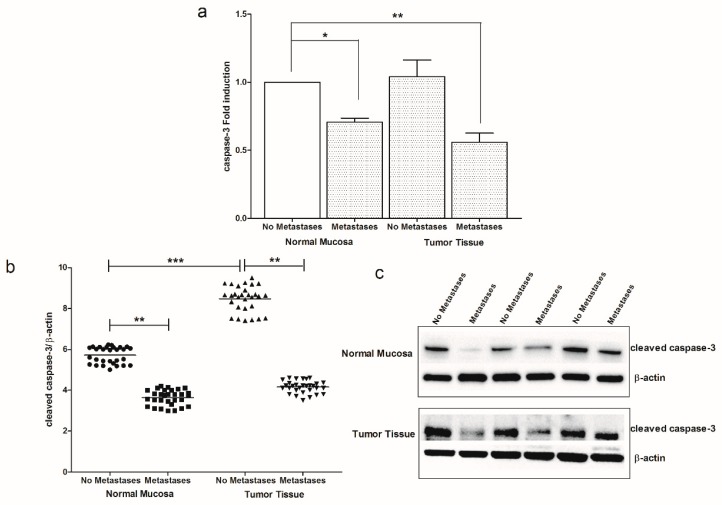
(**a**) Caspase-3 gene expression levels in intestinal tissue of no metastases (*n* = 29 patients) and with metastases patients (*n* = 30 patients) detected in both normal mucosa and tumor tissue. Data, expressed as mean value ± SD, are presented as fold induction compared to normal mucosa of patients without metastases. (**b**) Dot plots graph of cleaved caspase-3 protein values detected in our patients groups. *p < 0.05, **p < 0.02, ***p < 0.001 indicate statistically significant differences (one-way analysis of variance with Dunnett’s and Tukey’s multiple comparison test, where appropriate). (**c**) Representative Western blot bands of cleaved caspase-3 and β-actin proteins.

Moreover, in our CRC tissue samples, the apoptotic process has been further studied, investigating the levels of gene and protein expression of caspase-3. Lower levels of caspase-3 mRNA and protein were found in CRC patients with metastasis, both in normal mucosa and tumor tissue (Figure 7a–c).

## 3. Discussion

CRC patients with synchronous metastasis at first diagnosis and those patients developing metastasis during the course of disease account often for most cancer-related deaths, because of limited treatment options [18,19,20,21,24]. Optimal treatment has not yet been defined and the majority of patients with metastatic CRC are still being managed with palliative care.

In light of this issue, understanding the molecular mechanisms and the factors that regulate the formation of metastasis is crucial in developing effective therapies for metastatic cancer.

Among the bioactive molecules playing a role in the regulation of signaling pathways involved in tumor growth and progression, there is the CB1 protein, a G-protein-coupled transmembrane receptor, found not only predominantly in the central nervous system, but also in most peripheral tissues including immune cells and the gastrointestinal tract [25].

Here, we detected a low expression of CB1 receptor in metastatic CRC patients resulting in an alteration of its downstream signaling. CB1 receptor expression levels affect the intracellular levels of MAPK-p38 and ERK protein, downstream pathways dependent from CB1 receptor action. Literature data demonstrate that the antitumor effects of CB1 receptor occur via induction of MAPK pathways, such as MAPK-p38 and ERK1/2, and through the inhibition of the Akt-pathway, which in turn activate proapoptotic and inactivate antiapoptotic proteins [6,9,26,27].

Consistently with this hypothesis, we demonstrate, in metastatic CRC, an up-regulation of p-Akt, and the subsequent inhibition of cancer cell death, confirmed by a low gene and protein expression of Bax and caspase-3.

The direct participation of MAPK pathway in the antitumor action of cannabinoid receptors has been clearly demonstrated in pancreatic and hepatic cancer cells [8,28]. The cannabinoid-evoked apoptosis via CB1 receptor induction is stimulated by cell autophagy, known to be a cytoprotective mechanism leading to cell death [29,30]. It has also been reported that autophagy blockade prevents cannabinoid-induced apoptosis [31].

CB1 receptor expression has already been demonstrated to be correlated with distant metastasis in CRC [32]. However, the mechanisms underlying the transcriptional regulation of the CB1 receptor have not been clearly investigated.

The present study demonstrates that CB1 receptor down-regulation is associated with molecular changes, due to the switching off or on of the downstream pathways linked to this receptor function.

These signaling routes also result altered in intestinal normal mucosa which surrounding neoplasia, suggesting that tumor development and progression are certainly affected by the molecular defects that arise first in normal tissue. Therefore, intestinal normal mucosa from CRC patients provides a realistic picture of the molecules involved in tissue malignant transformation.

## 4. Materials and Methods

### 4.1. Patients

Fifty-nine consecutive patients with histologically proven colorectal cancer were recruited by the Surgery Division of our Institute. All patients were invited to provide an informed consent to take part in the study. At surgery, samples of mucosa, taken from macroscopically normal areas of intestine, at 10 cm from the neoplasia and cancer tissue, were obtained for each subject and stored at −80 °C until assayed.

The study was conducted in accordance with the Helsinki Declaration and approved by the Ethical Committee of IRCCS “S. de Bellis”, Castellana Grotte (Bari, Italy, number code: 32/CE/DE BELLIS, 27 October 2016).

### 4.2. Gene Expression Analysis

CB1 receptor, caspase-3, MAPK p38α, bax and bcl2 gene expression levels were evaluated in normal mucosa and cancer using the quantitative PCR (qPCR) method with SYBR1 green dye. Colorectal tissue samples were re-suspended in 0.3 mL of phosphate buffered saline and used for RNA extraction. Total cell RNA was extracted using Tri-Reagent (Mol. Res. Center Inc., Cincinnati, OH, USA), following the manufacturer’s instruction. About 2 μg total cell RNA was used for cDNA synthesis. Reverse transcription (RT) was carried out in 20 μL of the final volume at 42 °C for 30 min, using the iScript Advanced cDNA Synthesis Kit (Bio-Rad, Milan, Italy). Real-time PCRs were performed in 20 µL of a final volume containing 2 µL of cDNA, master mix with SYBR Green (iQ SYBR Green Supermix, Bio-Rad, Milan, Italy) and sense and antisense primers for the CB1 receptor, caspase-3, MAPK p38α, bax, bcl2, and the β-actin gene (Table 2). The β-actin gene was used as an internal control and was chosen as a reference gene.

Real-time PCRs were carried out in a CFX96 Real-Time PCR Detection System (Bio-Rad, Milan, Italy) using the following protocol: 45 cycles at 95 °C for 3 min, 95 °C for 10 s, 55 °C for 30 s followed by a melting curve step at 65–95 °C with a heating rate of 0.5 °C per cycle for 80 cycles. Relative quantification was done using the ∆∆Ct method.

### 4.3. Western Blotting

Total protein extracts were obtained treating each tissue sample with total lysis buffer (Pierce Ripa buffer, Thermo Scientific, Rockford, IL, USA) supplemented with protease and phosphatase inhibitors (Thermo Scientific, Rockford, IL, USA). After homogenization and centrifugation at 14,000 rpm for 15 min at 4 °C, the protein concentration was measured by a standard Bradford assay (Bio-Rad, Milan, Italy). Aliquots of 50 µg of total protein extracts from each sample were denaturated in 5× Laemmli sample buffer and loaded into 4–12% pre-cast polyacrylamide gels (Bio-Rad, Milan, Italy) for western blot analysis. Cannabinoid receptor I (Abcam, Cambridge, UK), cleaved caspase-3 (Asp175), Bax, ERK1/2, p-ERK1/2 (Thr202/Tyr204), p38α MAPK, p-p38 (Thr180/Tyr182) MAPK, Akt, p-Akt (Thr308), p-Akt (Ser473), β-actin (Cell Signaling Technology, Beverly, MA, USA) and Bcl2 (Santa Cruz Biotechnology, Santa Cruz, CA, USA), were used as primary antibodies. After overnight incubation, the membranes were incubated with a horseradish peroxidase-conjugated secondary antibody (Bio-Rad, Milan, Italy). The proteins were detected by chemiluminescence (ECL, Thermo Scientific, Rockford, IL, USA) and each protein-related signal was obtained using the Molecular Imager Chemidoc^TM^ (Bio-Rad, Milan, Italy) and normalized against β-actin protein expression.

### 4.4. Statistical Analysis

Data description was performed by using means ± SD. Then, data were analyzed using ANOVA with Dunnett’s and Tukey’s multiple comparison test, where appropriate. A *p* value ≤ 0.05 was considered as statistically significant.

## 5. Conclusions

Our data demonstrate that low expression of CB1 receptor in CRC positively affects the metastatic process, inhibiting apoptosis and deregulating the main signaling pathways at the basis of the receptor action. These observations contribute to the idea that drugs directed at regulating the endocannabinoid system through the induction of CB1 receptor, can be helpful in order to develop new anti-cancer therapies or improve existing ones.

## Figures and Tables

**Table 1:** Clinical and histopathological features of colorectal cancer patients with and without synchronous metastasis.

CRC Patients		
Variables	No Metastases (*n* = 29)	Metastases (*n* = 30)
Age	69.7 ± 15.2	68.3 ± 11
Sex		
Male	16	20
Female	13	10
Tumor Side ^a^		
Right	11	10
Left	18	20
Tumor Stage ^b^		
Stage I	5	2
Stage II	20	0
Stage III	3	18
Stage IV	1	10
Histological Grading		
Well-differentiated (G1)	3	0
Moderately-differentiated (G2)	16	16
Poorly-differentiated (G3)	10	14
Metastases Site		
Liver	0	12
Visceral lymphnodes	0	16
Bone	0	1
Lung metastases	0	1

^a^ Right side: hepatic flexure, cecum and ascending colon; Left side: descending colon, sigmoid and rectum. ^b^ Clinical staging performed using UICC System.

**Table 2:** Sequences of primers for gene expression analysis.

Gene	Primer
*CB1 receptor*	
Forward	5′-GGAGAACATCCAGTGTGGGG-3′
Reverse	5′-CATTGGGGCTGTCTTTACGG-3′
*caspase-3*	
Forward	5′-TGAGGCGGTTGTAGAAGAGTTT-3′
Reverse	5′-TTAACGAAAACCAGAGCGCC-3′
*MAPK p38α*	
Forward	5′-ACTCAGATGCCGAAGATGAAC-3′
Reverse	5′-GTGCTCAGGACTCCATCTCT-3′
*bax*	
Forward	5′-CAGGATGCGTCCACCAAGAA-3′
Reverse	5′-GCTCCCGGAGGAAGTCCAAT-3′
*bcl2*	
Forward	5′-GTGGAGGAGCTCTTCAGGGA-3′
Reverse	5′-AGGCACCCAGGGTGATG-CAA-3′
*β-actin*	
Forward	5′-AAAGACCTGTACGCCAACACAGTGCTGTCTGG-3′
Reverse	5′-CGTCATACTCCTGCTTGCTGATCCACATCTGC-3′

## Supplementary Materials

The following are available online at https://www.mdpi.com/2072-6694/11/5/708/s1, Figure S1: All Western blot figures include a dot plots graph showing the densitometry values of each sample (band) normalized to β-actin value.
