# Supplementary material for: Down-Regulation of Cannabinoid Type 1 (CB1) Receptor and its Downstream Signaling Pathways in Metastatic Colorectal Cancer
Source: Cancers (Basel). 2019 May 22;11(5):708. doi: 10.3390/cancers11050708 (PMC6562552; doi:10.3390/cancers11050708)
Supplement: Supplementary file 1 [file cancers-11-00708-s001.pdf]

Supplementary Material

Down-Regulation of Cannabinoid Type 1 (CB1) Receptor and its Downstream Signaling Pathways in Metastatic Colorectal Cancer

Valeria Tutino, Maria Gabriella Caruso, Valentina De Nunzio, Dionigi Lorusso, Nicola Veronese, Isabella Gigante, Maria Notarnicola and Gianluigi Giannelli

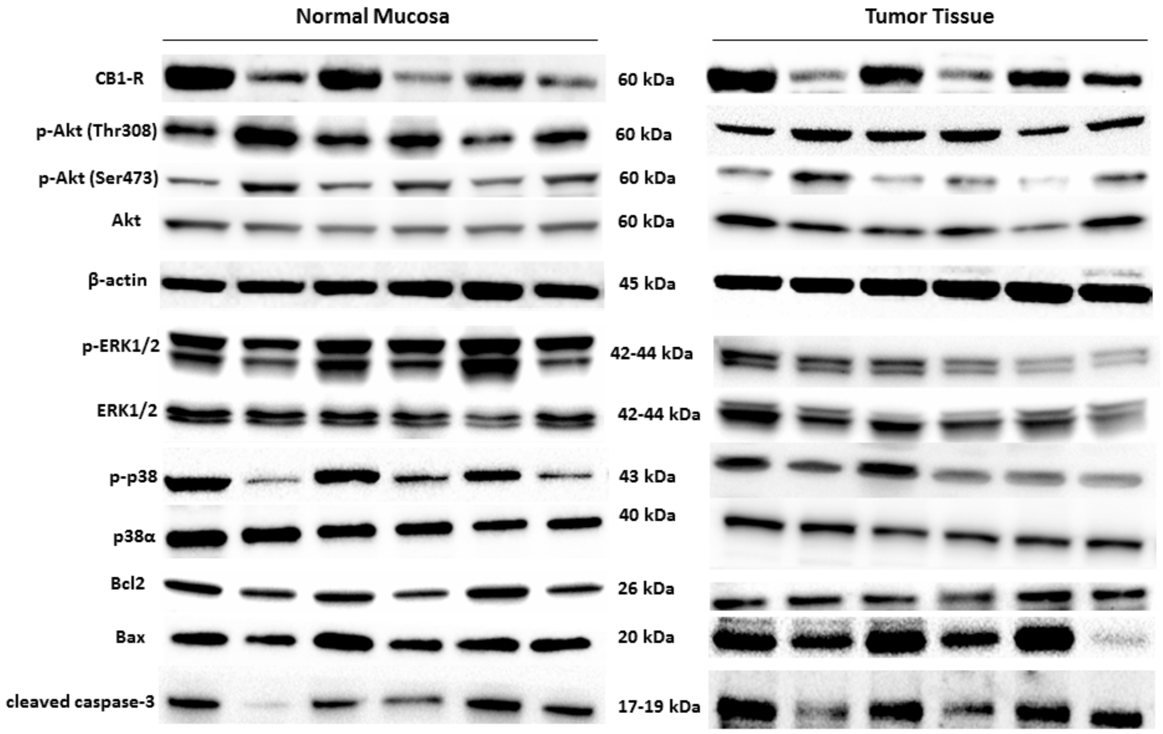

Figure S1. All Western blot figures include a dot plots graph showing the densitometry values of each sample (band) normalized to β-actin value.

© 2019 by the authors. Licensee MDPI, Basel, Switzerland. This article is an open access article distributed under the terms and conditions of the Creative Commons Attribution (CC BY) license (http://creativecommons.org/licenses/by/4.0/).
